# Dynamin Inhibitors Impair Endocytosis and Mitogenic Signaling of PDGF

**DOI:** 10.1111/tra.12061

**Published:** 2013-03-12

**Authors:** Łukasz Sadowski, Kamil Jastrzębski, Yannis Kalaidzidis, Carl-Henrik Heldin, Carina Hellberg, Marta Miaczynska

**Affiliations:** 1 Laboratory of Cell Biology, International Institute of Molecular and Cell BiologyWarsaw, Poland; 2 Max Planck Institute of Molecular Cell Biology and GeneticsDresden, Germany; 3 Science for Life Laboratory, Ludwig Institute for Cancer Research Ltd., Uppsala UniversityUppsala, Sweden; 4 School of Biosciences, University of BirminghamBirmingham, UK

**Keywords:** dynamin inhibitors, endocytosis, PDGF, signaling, STAT3

## Abstract

Platelet-derived growth factor (PDGF) isoforms regulate cell proliferation, migration and differentiation both in embryonic development and adult tissue remodeling. At the cellular level, growth-factor signaling is often modulated by endocytosis. Despite important functions of PDGF, its endocytosis remains poorly studied, mainly for lack of tools to track internalized ligand by microscopy. Here, we developed such a tool and quantitatively analyzed internalization and endosomal trafficking of PDGF-BB in human fibroblasts. We further show that PDGF can be internalized in the presence of dynamin inhibitors, arguing that both dynamin-dependent and dynamin-independent pathways can mediate PDGF uptake. Although these routes operate with somewhat different kinetics, they both ultimately lead to lysosomal degradation of PDGF. Although acute inhibition of dynamin activity only moderately affects PDGF endocytosis, it specifically decreases downstream signaling of PDGF via signal transducer and activator of transcription 3 (STAT3). This correlates with reduced expression of *MYC* and impaired cell entry into S-phase, indicating that dynamin activity is required for PDGF-induced mitogenesis. Our data support a general view that the components governing endocytic trafficking may selectively regulate certain signaling effectors activated by a growth factor.

Endocytic internalization and trafficking play important roles in the regulation of signaling by various receptor types, including receptor tyrosine kinases (RTKs) 1–3. Internalization of receptors can occur via different structures formed from the plasma membrane, such as clathrin-coated vesicles or caveolae (which require the large GTPase dynamin for pinching off), or dynamin- and clathrin-independent carriers (CLICs) 4–6. Different internalization pathways may direct the receptor to recycling or degradation, thus leading to sustained signaling or its termination, respectively 7–10. Moreover, many receptors remain active after internalization into endosomes which enables compartmentalization and amplification of signals, as well as delivery of signaling complexes into specific intracellular sites 2.

Platelet-derived growth factor (PDGF) is a family of isoforms regulating cell proliferation, migration and differentiation 11. They are required for embryonic development and for adult tissue remodeling, in particular vascular development, angiogenesis and wound healing 12. PDGF isoforms act through two related RTKs, platelet-derived growth factor receptor α (PDGFRα) and PDGFRβ which homo- and heterodimerize upon ligand binding. All PDGFs are disulfide bond-linked dimers of four polypeptides (A, B, C, D) which bind specific receptor dimers. The PDGF-BB homodimer binds all receptor combinations *in vitro* 13, although *in vivo* its action via PDGFRβ homodimers is particularly important 12. Upon ligand-induced dimerization, receptor autophosphorylation creates docking sites for downstream effectors which initiate signaling pathways, involving Ras/extracellular signal-regulated kinase (ERK) mitogen-activated protein kinase (MAPK), phosphatidylinositol 3-kinase/AKT, *src* kinase and signal transducer and activator of transcription (STAT), eventually altering gene expression 12. Moreover, ligand binding stimulates receptor internalization, resulting in lysosomal degradation of PDGF-PDGFR complexes 13, 14. Before reaching its final destination, a certain amount of the receptor remains active intracellularly and is capable to propagate signaling 15, 16. PDGF concentration was shown to regulate the physiological response of cells by a differential activation of certain signaling effectors, with low ligand amounts inducing cell migration and high amounts resulting in proliferation 7. In the case of epidermal growth factor (EGF), ligand concentration was reported to dictate the internalization routes of the receptor 9. By analogy, it was proposed that different modes of internalization induced by low- or high-PDGF concentration may switch cellular responses, although this argument was based on indirect evidence without visualizing PDGF endocytosis 7.

In contrast to the well-studied EGF, no commercial tools to visualize PDGF in cells are available, such as labeled ligands or antibodies suitable for indirect immunofluorescence staining. Tracking of internalized PDGF in fluorescence microscopy has been a challenge because of its highly adhesive properties. *In vivo*, bioavailability of PDGF is maintained by binding to the extracellular matrix via a C-terminal basic retention motif 17 and such PDGF retention plays important physiological roles, e.g. in pericyte recruitment and renal function in mice 18. However, these molecular properties of PDGF cause its high adhesion to glass or plastic under physiological pH which results in extreme extracellular background when using directly labeled ligands, precluding any quantitative analysis. For this reason, most previous reports visualized PDGFRs by indirect immunofluorescence to infer conclusions about ligand trafficking, although this approach cannot distinguish between ligand-bound or free receptors 19–22.

In this work, we developed a tool to track internalized PDGF-BB by confocal microscopy in the absence of extracellular background. We used it to quantitatively analyze endocytic trafficking of PDGF-BB and its impact on activation of individual signaling effectors. We demonstrate that PDGF-BB is internalized via dynamin-dependent and dynamin-independent pathways, and that dynamin activity is crucial for the activation of STAT3, induction of *MYC* expression and DNA synthesis to initiate cell proliferation.

## Results and Discussion

### Visualization of PDGF endocytosis with a novel assay

**Figure 1:**
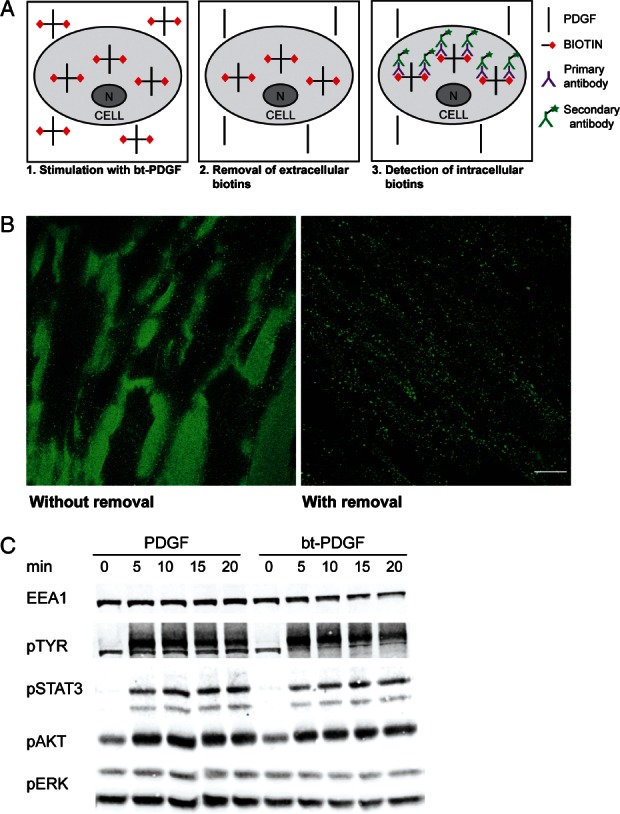
Microscopical assay to detect internalized PDGF A) Schematic of bt-PDGF detection. Cells are stimulated with bt-PDGF 1. Following internalization, biotins on extracellular bt-PDGF are removed by a reducing agent 2 and only intracellular bt-PDGF is detected by anti-biotin antibodies 3. B) Images of cells stimulated with 100 ng/mL bt-PDGF: before (left) and after (right) removal of extracellular biotins. Scale bar 20 µm. C) Activation of PDGFR and its downstream effectors upon stimulation of cells with 100 ng/mL unlabeled PDGF or bt-PDGF, visualized by immunoblotting with phospho-specific antibodies against tyrosine (pTYR; for PDGFR phosphorylation), STAT3, AKT and ERK.

To track internalized PDGF-BB (referred to as PDGF in this study for simplicity) by microscopy and to eliminate extracellular background observable upon its direct labeling with fluorescent dyes, we conjugated PDGF to biotin using a linker cleavable by reducing agents. The rationale behind it was to stimulate cells with the biotinylated PDGF-BB (bt-PDGF), followed by the removal of extracellular biotin molecules with a reducing agent and detection of internalized PDGF with anti-biotin antibodies (Figure 1A). Throughout our study, we used human foreskin fibroblasts CCD-1070Sk with high levels of endogenous PDGFRβ. When bt-PDGF was applied to cells, following fixation and staining with anti-biotin antibodies, high extracellular background was predominantly visible on the coverslip in addition to the weak intracellular staining (similar images were obtained upon direct labeling of PDGF with fluorescent dyes, Figure S1A). However, when cells were incubated on ice with glutathione to cleave-off extracellular biotin labels after stimulation, followed by fixation and anti-biotin staining, the background was removed and internalized PDGF was clearly visible by confocal microscopy in intracellular vesicular structures (Figure 1B). We carefully optimized the procedure of PDGF biotinylation to avoid excessive labeling which was inhibitory for the PDGF activity (data not shown). Throughout our study, we used preparations containing three to five biotins per PDGF dimer, as determined by mass spectrometry analysis. This degree of labeling did not perturb the signaling activity of bt-PDGF, which induced tyrosine phosphorylation of the receptor and activation of STAT3, AKT, ERK1/2 to an extent comparable with the unlabeled ligand (Figure 1C). Thus, reversible biotinylation proved to be an efficient method of PDGF labeling for fluorescence microscopy.

**Figure 2:**
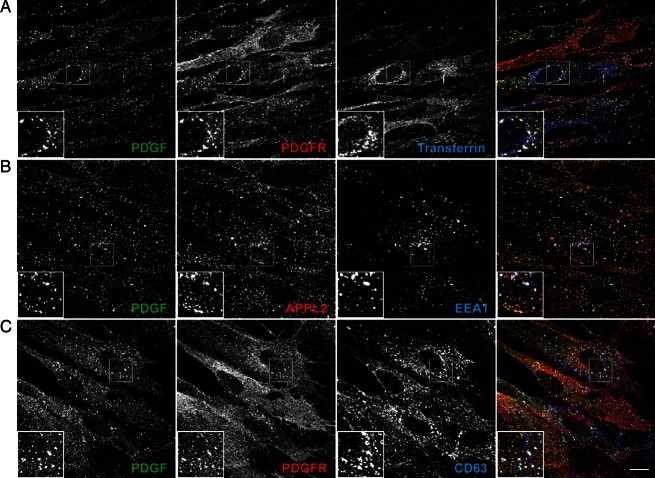
Endosomal trafficking of PDGF Cells were stimulated with: 100 ng/mL bt-PDGF and 20 µg/mL transferrin-Alexa647 for 20 min (A); bt-PDGF for 20 min (B) or 40 min (C). Cells were immunostained for biotin (bt-PDGF), PDGFRβ, APPL2, EEA1 or CD63. Scale bar 20 µm.

Further confirming the specificity of bt-PDGF labeling, we detected very extensive overlap (>80%) between the ligand and PDGFRβ at various times of internalization (Figure 2A and data not shown). Within 20 min, bt-PDGF was largely colocalized with the markers of early endosomes, early endosome antigen 1 (EEA1) and adapter protein containing PH domain, PTB domain and leucine zipper motif 2 (APPL2) 23, 24 (Figure 2B). Transferrin internalized for 30 min as a recycling marker 25 exhibited limited colocalization with bt-PDGF (Figure 2A), in agreement with previous findings that PDGF-PDGFR complexes are predominantly degraded rather than recycled under physiological conditions 21. Consistently, PDGF and PDGFRβ were found in CD63-positive late endosomes within 40 min of stimulation 26 (Figure 2C).

### Quantitative analysis of PDGF trafficking

**Figure 3:**
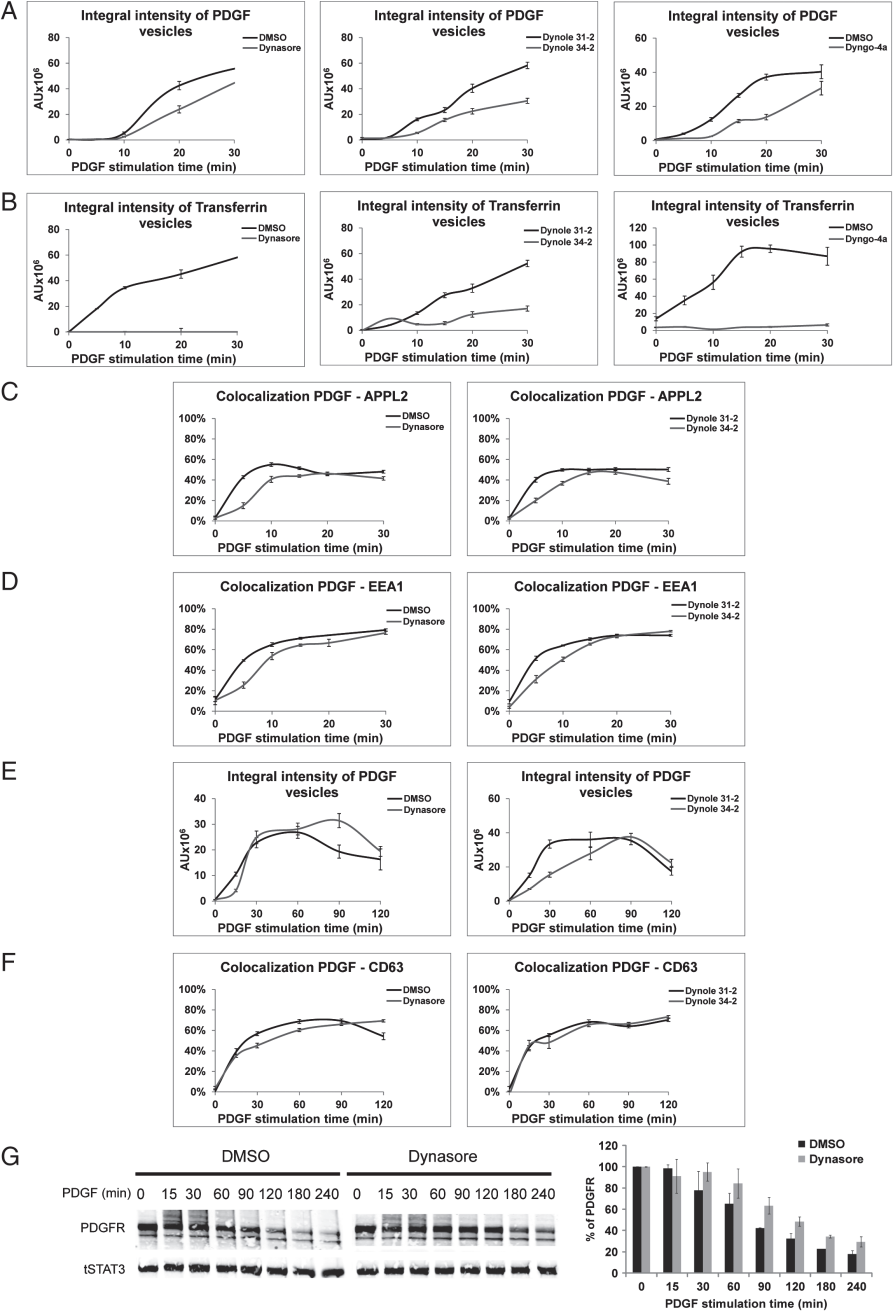
Quantitative analysis of PDGF trafficking and PDGFR degradation upon inhibition of dynamin A–F) Cells stimulated with bt-PDGF (100 ng/mL) and transferrin-Alexa647 in the presence of indicated inhibitors were fixed and immunostained for biotin (bt-PDGF), EEA1, APPL2 or CD63. Acquired images were analyzed to determine: total intracellular accumulation of PDGF (A, E) or transferrin (B), expressed as integral fluorescence of all detectable vesicles; or colocalization of bt-PDGF with APPL2 (C), EEA1 (D) or CD63 (F). Error bars are SEM. G) PDGFRβ degradation upon dynamin inhibition. Lysates of cells stimulated with 50 ng/mL PDGF in the presence of dynasore or DMSO for up to 240 min were immunoblotted for PDGFRβ and STAT3 (left panel shows a representative blot). Signals of PDGFRβ were quantified and normalized against signals of total levels of STAT3 (right chart shows average values of two independent experiments, error bars are SD).

Determination of kinetics of endocytosis of growth factors and their receptors is required to understand the global properties of their signaling 27, 28. Therefore, we quantitatively analyzed endocytic trafficking of PDGF-PDGFR complexes as a prerequisite to elucidate its impact on PDGF signaling. After stimulation with 100 ng/mL bt-PDGF for various time periods, we quantified the accumulation of bt-PDGF vesicles over time, as well as their colocalization with PDGFRβ and endosomal markers EEA1, APPL2 and CD63 using previously validated MotionTracking software 29, 30 (Figure 3). In general, the internalization kinetics of bt-PDGF (Figure 3A) is similar to that previously reported using radiolabeled ligand 31, thus additionally validating our newly developed tool.

To determine the contribution of dynamin-dependent endocytosis to PDGF internalization, we decided to perform parallel analyses in cells treated with inhibitors of dynamin GTPase activity. Use of chemical inhibitors has the advantage of acute interference with the dynamin activity, without affecting its presence and potential interactions in the cells. The short-term treatment with inhibitors minimizes any indirect, compensatory effects which may take place upon long-term reduction of dynamin levels in the cells. Indeed, testing three human fibroblast lines (CCD-1070Sk, HFF and MRC-5), we were unable to silence the expression of dynamin 2 (a predominant form of dynamin in these and most other cell types) efficiently enough to observe a robust block of transferrin uptake (data not shown). This is consistent with the results of dynamin knock-out studies, arguing that even very low levels of dynamin 2 in the brain, upon knock-out of dynamins 1 and 3, can support neuronal functions 32.

For our analysis, we selected three inhibitors: dynasore 33, dynole 34–2 34 and dyngo-4a 35 which belong to different chemical classes but exhibit an inhibitory activity towards the GTPase allosteric site (GAS) of both dynamins 1 and 2. Indeed, all three inhibitors efficiently blocked uptake of transferrin, a typical cargo of dynamin-dependent endocytosis, upon 30-min pretreatment of cells (Figure 3B). Initially, we also tried to employ other dynamin inhibitors, such as Iminodyn-22 targeting the GAS 36 or MiTMAB and OcTMAB which target the PH domain of dynamin 37, 38. However, Iminodyn-22 treatment led to unspecific effects (a complete inhibition of PDGFR phosphorylation concomitant with blocked internalization of PDGFR and transferrin; Figure S1B,C), whereas MiTMAB and OcTMAB showed rapid cytotoxicity in a broad range of concentrations (data not shown). These inhibitors were thus excluded from further analysis.

The three selected dynamin inhibitors, dynasore, dynole 34–2 and dyngo-4a, caused a moderate (22–47%) reduction of bt-PDGF uptake after 30 min (Figure 3A), while in the same cells the block of transferrin uptake was much stronger (more than 90% for dynasore and dyngo-4a; 70% for dynole 34–2; Figure 3B). These data demonstrate that PDGF-BB can be internalized, although less efficiently, upon inhibition of dynamin activity. This argues that, at least when applied at 100 ng/mL concentration, PDGF can be taken up via dynamin-independent carriers.

Within the first 30 min of internalization, PDGF progressively accumulates in early endosomes. APPL endosomes, bearing APPL1 and APPL2 proteins, are a subpopulation of endosomes involved in early trafficking of EGF 23, 39. Here, we show that APPL endosomes (visualized by APPL2 staining, as APPL1 is poorly expressed in CCD-1070Sk cells; data not shown) also participate in the transport of PDGF, as up to 50% of detectable bt-PDGF accumulates in this compartment between 10 and 30 min of stimulation (Figure 3C). These data add PDGF-BB and PDGFR to the list of cargo known to be transported via APPL endosomes. In parallel, up to 80% of detectable bt-PDGF colocalizes with EEA1 within 20–30 min of stimulation (Figure 3D). Inhibition of dynamin activity had only a limited impact on PDGF sorting into APPL and EEA1 endosomes, delaying cargo delivery to these compartments within the first 5–10 min of stimulation, without affecting the maximal accumulation at 30 min.

To investigate further trafficking of PDGF towards late endosomes, we measured PDGF colocalization with CD63 within 2 h of stimulation. In control cells treated with dimethyl sulfoxide (DMSO), PDGF reached maximal intracellular accumulation at 1 h, followed by a slow decay reflecting its progressive degradation (Figure 3E,G). After 2 h of stimulation with 50 ng/mL PDGF, about 60% of the initial receptor amount was degraded when assessed biochemically (Figure 3G). This value is comparable with the 40% downregulation of ligand binding sites after 2 h of stimulation with 50 ng/mL radiolabeled PDGF-BB, reported in a classical quantitative study using endothelial cells overexpressing PDGFR 31. We further observed that the degradation kinetics was altered in dynasore-treated cells. Despite an initial delay in internalization, the maximal accumulation of PGDF was not decreased but clearly prolonged, indicating a retarded initiation of degradation (Figure 3E). This conclusion was supported by biochemical data, demonstrating delayed degradation of PDGFRβ upon dynasore treatment (Figure 3G). In turn, changes induced by dynole 34–2 involved slower intracellular accumulation of PDGF within 90 min without affecting its subsequent decay (Figure 3E). However, an inactive negative control (dynole 31–2) also altered the kinetics of PDGF accumulation in comparison with DMSO, indicating that this class of molecules may have some unspecific side-effects upon longer application (consistent with our observations of their increasing toxicity on CCD-1070Sk cells starting from 1 to 2 h of incubation; data not shown). In spite of these differences, neither dynasore nor dynole affected sorting of PDGF to late endosomes, as the proportion of PDGF colocalizing with CD63 was unaltered (Figure 3F).

### Dynamin-dependent changes in PDGF signaling

**Figure 4:**
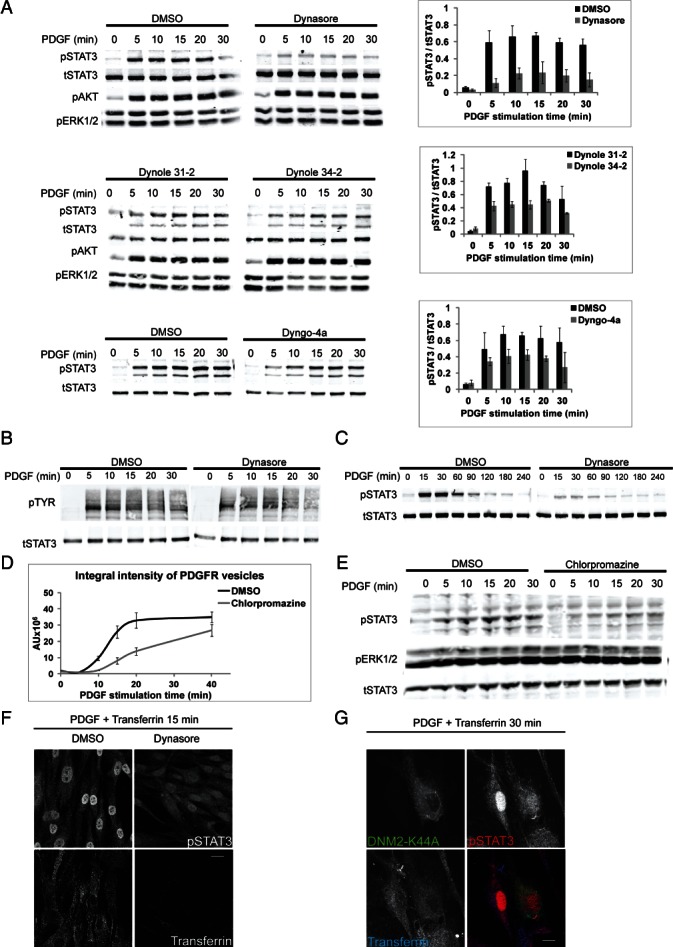
Dynamin inhibitors impair PDGF-induced activation of STAT3 A) Lysates of CCD-1070Sk cells stimulated with 50 ng/mL PDGF in the presence of indicated inhibitors were immunoblotted for phospho-STAT3, phospho-AKT, phospho-ERK1/2 and total STAT3 (left panels show representative blots). Signals of phospho-STAT3 were quantified and normalized against signals of total STAT3 (right charts show average values of three independent experiments, error bars are SD). B) Activation of PDGFR upon stimulation of cells with 50 ng/mL PDGF in the presence of dynasore or DMSO, visualized by immunoblotting with pTYR antibodies (total STAT3 as loading control). C) Long-term dynamics of PDGF-induced STAT3 activation. Lysates of cells stimulated with 50 ng/mL PDGF in the presence of dynasore or DMSO for up to 240 min were immunoblotted for phospho-STAT3 and total STAT3, as indicated. D) Kinetics of PDGF uptake upon inhibition of endocytosis with chlorpromazine. Cells stimulated with bt-PDGF (100 ng/mL) in the presence of chlorpromazine or DMSO for various time periods were fixed and immunostained for biotin (bt-PDGF). Acquired images were analyzed to determine total intracellular accumulation of PDGF expressed as integral fluorescence of all detectable vesicles. E) Lysates of cells stimulated with 50 ng/mL PDGF in the presence of chlorpromazine or DMSO were immunoblotted for phospho-STAT3, phospho-ERK1/2 and total STAT3. F) Immunofluorescence analysis of phospho-STAT3 nuclear translocation in cells stimulated with PDGF (50 ng/mL) and transferrin-Alexa647 for 15 min in the presence of dynasore or DMSO. G) Immunofluorescence analysis of phospho-STAT3 nuclear translocation in cells overexpressing GFP-tagged K44A mutant of dynamin 2 (DNM2-K44A) and stimulated with PDGF (50 ng/mL) and transferrin-Alexa647 for 30 min. F and G) Scale bar 20 µm.

Having established that PDGF can be internalized in dynamin-dependent and dynamin-independent manners in human fibroblasts, we analyzed whether any of these pathways may be preferentially linked to the activation of certain signaling effectors of PDGF. We tested the levels of activated (phosphorylated) ERK1/2 MAPK, AKT and STAT3 upon PDGF stimulation, with or without dynamin inhibitors. Under control conditions, 50 ng/mL PDGF induced robust activation of AKT and STAT3, but not of ERK1/2 which were already activated in serum-starved cells (Figure 4A). In turn, PDGF at a lower concentration (5 ng/mL) does not induce STAT3 phosphorylation, while still activating AKT (Figure S1D). Strikingly, all three dynamin inhibitors decreased STAT3 phosphorylation following PDGF stimulation, whereas phosphorylation of AKT and ERK1/2 remained unaltered (Figure 4A). The latter result is in agreement with the observations in mouse fibroblasts with conditional knock-out of dynamins 1 and 2, where no changes in the activation of ERK1 and AKT were reported upon EGF stimulation 40.

We further verified that reduced STAT3 activation does not result from decreased ligand-stimulated autophosphorylation of PDGFR upon inhibition of dynamin activity (Figure 4B). Moreover, we excluded that STAT3 activation is only delayed under these conditions, because no late activation of STAT3 was observed in cells treated with dynasore for 4 h (Figure 4C). Furthermore, the same selective effect of dynasore on the activation of STAT3 but not of ERK or AKT was confirmed in another human cell line of fetal lung fibroblasts MRC-5 (Figure S1E).

Dynamins are multifunctional GTPases, with a range of cellular activities in addition to endocytosis 41. Therefore, the observed effects of dynamin inhibitors raised a question of whether impaired STAT3 activation results from partial inhibition of PDGF endocytosis or from other endocytosis-independent functions of dynamins. To address this issue, we used chlorpromazine as a broad inhibitor of predominantly clathrin-dependent endocytosis 42. The initial rate of PDGF internalization was reduced upon chlorpromazine treatment (Figure 4D) in a manner similar to dynasore. Impaired endocytosis of PDGF correlated with lower phosphorylation of STAT3, whereas ERK activation remained unchanged (Figure 4E). These data indicate that internalization of PDGF is linked to STAT3 activation. Furthermore, it is unlikely that the effects of dynamin inhibitors on PDGF-induced STAT3 phosphorylation are exclusively due to the inhibition of non-endocytic functions of dynamin.

Activation of STAT3 leads to its nuclear translocation where it acts as a transcription factor 43. In agreement with the biochemical data, dynasore treatment reduced the nuclear translocation of STAT3 following PDGF stimulation (Figure 4F). Importantly, expression of the dominant-negative K44A mutant of dynamin 2 44 impaired nuclear accumulation of STAT3 in a similar manner (Figure 4G), independently confirming the results obtained with dynamin inhibitors. Cumulatively, our data indicate that STAT3 activation by PDGF requires dynamin activity and high ligand concentration.

### Impairment of PDGF-induced gene expression and DNA synthesis upon inhibition of dynamin

**Figure 5:**
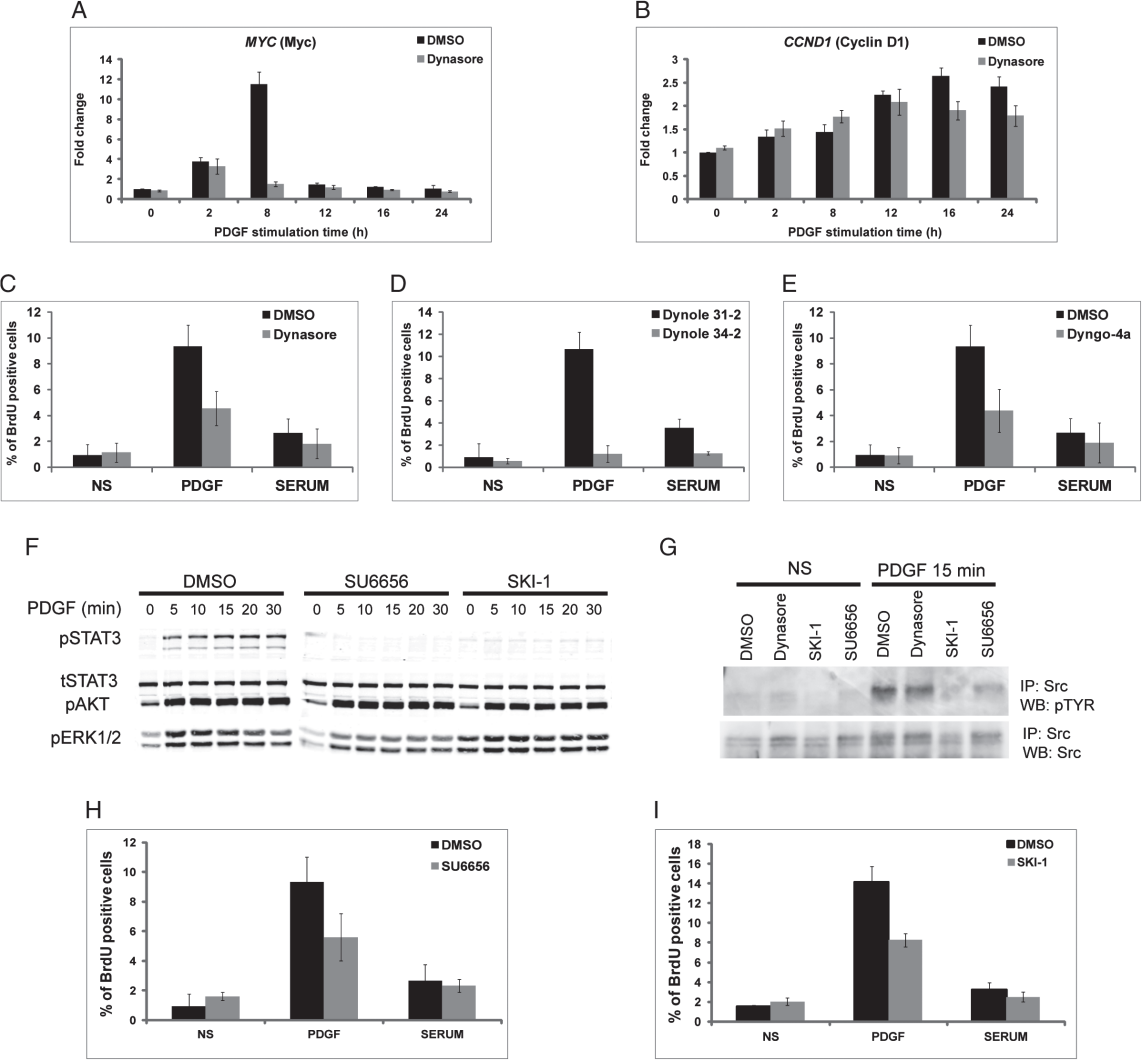
Impairment of PDGF-induced gene expression and DNA synthesis upon inhibition of dynamin Real-time PCR analysis of PDGF-induced expression of *MYC* (A) and *CCND1* (Cyclin D1 (B) in the presence of dynasore or DMSO. C–E) DNA synthesis measured by BrdU incorporation in cells stimulated with 50 ng/mL PDGF, 10% serum or left non-stimulated (NS) in the presence of the indicated inhibitors of dynamin. Charts present a percentage of BrdU-positive cells calculated based on a total number of cells measured by 4′,6-diamidino-2-phenylindole (DAPI) staining. Error bars are SEM. F) PDGF-induced signaling in cells treated with the indicated *src* inhibitors and stimulated with PDGF. Lysates were immunoblotted for phospho-STAT3, phospho-AKT, phospho-ERK1/2 and total STAT3. G) PDGF-induced activation of *src* in the presence of the indicated inhibitors was analyzed by immunoprecipitation from lysates of cells non-stimulated (NS) or stimulated with 50 ng/mL PDGF. Immunoprecipitates were blotted for pTYR and total *src*. H–I) DNA synthesis measured by BrdU incorporation in cells stimulated with 50 ng/mL PDGF, 10% serum or left non-stimulated (NS) in the presence of the indicated *src* inhibitors. The results were analyzed as in (C–E).

Nuclear STAT3 binds to the promoters of multiple genes 45, with *MYC* being one of its key targets required for PDGF-induced mitogenesis 46, 47. We therefore tested how inhibition of dynamin activity during PDGF stimulation affected expression of *MYC* and *CCND1* (encoding cyclin D1) as well as DNA synthesis. Owing to possible unspecific effects and the cellular toxicity of dynamin inhibitors upon prolonged application, we shortened the treatment of cells with an inhibitor and PDGF. Of note, short pulses of PDGF (30–60 min) induced the first phase of mitogenic signaling as efficiently as continuous exposure to the ligand for 8 h 47. In our protocol, cells were preincubated for 30 min with an inhibitor, followed by 1 h incubation in the presence of PDGF. Cells were subsequently washed in PBS to remove excess ligand and kept in an inhibitor- and PDGF-free medium for up to 23 h. We observed a robust 11-fold induction of *MYC* expression 8 h after addition of PDGF (Figure 5A), consistent with previous reports 46, 48. This induction was completely abolished when dynasore was present during the incubation with PDGF. Induction of *CCND1* expression upon PDGF treatment was relatively low (reaching 2.5-fold at 16 h) but dynasore reduced it at 16 and 24 h after growth factor incubation (Figure 5B). Consistently, analyzing the protein levels of cyclin D1 by immunostaining, we observed its impaired nuclear accumulation in cells treated with dynasore (Figure S2). In spite of certain variability in the intensity of cyclin D1 staining between individual control cells, the inhibitory effect of dynasore was relatively uniform and visible in the majority of cells in the population.

Next, we investigated the effects of dynamin inhibitors on DNA synthesis, measured by 5-bromo-2'-deoxyuridine (BrdU) incorporation. We confirmed that addition of PDGF, but not of 10% serum, for 1 h was sufficient to promote cell entry into S-phase (Figure 5C–E). Strikingly, all three inhibitors (dynasore, dynole 34–2 and dyngo-4a) reduced the number of BrdU-positive cells when co-applied together with PDGF (Figure 5C–E). This implies that PDGF-induced mitogenesis via activation of STAT3 and MYC requires dynamin activity at the time of the PDGF application.

At a mechanistic level, PDGF-stimulated STAT3 activation was reported to depend on *src* kinase activity 49, 50. Indeed, two *src* inhibitors, SU6656 and SKI-1, abrogated PDGF-induced STAT3 phosphorylation in CCD-1070Sk fibroblasts (Figure 5F). However, in contrast to SKI-1 and SU6656, dynasore treatment did not affect *src* activation, arguing that *src* function is normal in cells with inhibited dynamin activity (Figure 5G). This indicates that, in agreement with the literature, *src* acts upstream of dynamin, phosphorylating it and stimulating its activity 51–55, therefore *src* inhibition impairs dynamin function and dynamin-dependent STAT3 signaling. Consistently, *src* inhibition by SU6656 or SKI-1 decreased BrdU incorporation upon PGDF stimulation, although somewhat less potently than dynamin inhibitors (Figure 5H,I). These quantitative differences may be explained by a broad range of dynamin functions, including its role in cytokinesis 56–60 which can be also affected by the use of dynamin inhibitors, in addition to the block of dynamin-mediated endocytosis.

In summary, our detailed evaluation of endocytic trafficking of PDGF-PDGFR revealed a significant contribution of dynamin-independent route(s) to the internalization of this complex. This may represent a physiological phenomenon or a compensatory mechanism induced by dynamin inhibition. Importantly, at least upon dynamin inhibition, such independent mechanisms can efficiently mediate uptake of PDGF (at later time-points reaching the control levels), although they operate slower at early times of stimulation (30–60 min). In spite of differences in the absolute amounts of internalized PDGF, its sorting to early and late endosomes was unaffected by dynamin inhibitors. This argues that both dynamin-dependent and dynamin-independent routes direct PDGF to the same endosomal compartments. Even though these routes may be functionally equivalent for endosomal trafficking of PDGF, they differ with respect to its mitogenic signaling. Although we cannot formally exclude that an endocytosis-independent activity of dynamin is also required for mitogenic signaling of PDGF, we propose that dynamin-mediated internalization of PDGF is coupled to STAT3 activation. This model is supported by our observations that chlorpromazine, an endocytosis inhibitor believed to act via interactions with membrane lipids and/or cytoskeletal regulators 42, impairs both PDGF uptake and PDGF-induced STAT3 phosphorylation.

We further propose that STAT3 activation as a key early event in PDGF mitogenic signaling occurs at the plasma membrane in dynamin-containing domains and/or during early steps of dynamin-mediated uptake, largely before cargo delivery to early endosomes. Upon dynamin inhibition, delayed accumulation of PDGF in endosomes in amounts similar to the control (Figure 3E) cannot compensate for an early defect in STAT3 signaling (Figure 4C). This argues that if STAT3 activation occurs on endosomes in PDGF-stimulated fibroblasts, then it is functionally negligible. This is in contrast to other receptors signaling via STAT3, e.g. Met was shown to activate STAT3 at perinuclear endosomes which was required for the amplification of weak STAT3 signals (occurring only after 2 h of Met stimulation) 61. In the case of PDGF, we observed robust STAT3 activation starting from 5 min, which additionally indicates that this signaling event occurs at the plasma membrane and/or on early endocytic intermediates. A similar dynamin-dependent activation of STAT3 upon PDGF stimulation observed in human fetal lung fibroblasts MRC-5 (Figure S1E) underscores a more universal mode of PDGF signaling in fibroblasts of various origins. Moreover, a recent study dealing with EGF reported an analogous, selective effect of dynamin inhibition or depletion on STAT3 but not ERK signaling in two human carcinoma cell lines 62. Combined with our data, this may point to a general mechanism of coupling the dynamin activity to STAT3 signaling upon growth factor stimulation in different cell types.

Our data support the view that different internalization routes regulate not only ligand targeting to different plasma membrane domains and endocytic intermediates but also the uptake kinetics and amounts of internalized ligand. Dynamin-mediated uptake ensures fast and efficient internalization of PDGF which correlates with its mitogenic signaling (low PDGF concentrations do not trigger STAT3 activation and mitogenesis). The existence of alternative ways of internalization coupled to selective activation of downstream signaling effectors may be exploited by cells to sense a PDGF gradient and switch from migration (low PDGF) to proliferation (high PDGF) 7. Different internalization routes linked to distinct signaling outcomes were previously proposed for EGF, TGFβ or Wnt ligands 8–10. In the case of PDGF, dynamin activity appears to be a key factor governing both internalization of PDGF and its mitogenic signaling via the STAT3-MYC cascade.

## Materials and Methods

### Antibodies and chemicals

The following antibodies were obtained from commercial sources: goat anti-biotin (#B3640; Sigma-Aldrich); rabbit anti-PDGFRβ (sc-432), rabbit anti-*c-src* (sc-18; Santa Cruz Biotechnology); rabbit anti-cyclin D1 (#RB-010-P1; Thermo Scientific); mouse anti-EEA1 (mAb, #610457; BD Biosciences); mouse anti-phosphotyrosine (pTYR) (#05-321; Millipore). The following antibodies were purchased from Cell Signaling Technology: rabbit anti-phospho-ERK1/2 (p44/42 MAPK; Thr202/Tyr204) (mAb #4370), anti-phospho-AKT (Ser473) (mAb #9271), anti-phospho-STAT3 (Tyr705) (mAb #9145), mouse anti-ERK1/2 (mAb #9107), anti-AKT (mAb #2920), anti-STAT3 (mAb #9139). The mouse anti-CD63 antibody (mAb, #H5C6) developed by J.T. August/J.E.K. Hildreth and the mouse anti-BrdU (mAb, #G3G4) antibody developed by S.J. Kaufman were obtained from the Developmental Studies Hybridoma Bank developed under the auspices of the NICHD and maintained by The University of Iowa, Department of Biology, Iowa City, Iowa, USA. The following rabbit antibodies were previously described: anti-APPL2 63 and anti-EEA1 64. The following secondary antibodies used for immunofluorescence were from Invitrogen: Alexa Fluor 488-, 555- and 647-conjugated anti-goat, anti-mouse and anti-rabbit, as well as transferrin labeled with Alexa Fluor 647 (transferrin-Alexa647). Secondary donkey antibodies for western blot infrared detection: IRDye 800CW anti-mouse and IRDye 680LT anti-rabbit were from LI-COR Biotechnology. Dynamin inhibitors: dynasore was purchased from Merck; dynole 34–2, dynole 31–2 (an inactive control for dynole 34–2), dyngo-4a, Iminodyn-22 were from Ascent Scientific. Endocytosis inhibitor chlorpromazine hydrochloride, *src* kinase inhibitors SU6656 and SKI-1 were purchased from Sigma-Aldrich. The inhibitors were used in the following concentrations: 80 μM dynasore, 20 μM dynole 34–2, 20 μM dynole 31–2, 30 μM dyngo-4a, 11 μM Iminodyn-22, 30 μM chlorpromazine, 2.5 μM SU6656 and 2.5 μM SKI-1. DMSO (Bioshop Canada) served in an equivalent volume as a control for dynasore, dyngo-4a, Iminodyn-22, SU6656 and SKI-1, and as a solvent for all inhibitors and controls. PDGF-BB was purchased from PeproTech.

### Generation of bt-PDGF by reversible biotinylation

PDGF-BB was biotinylated with Sulfo-NHS-SS-Biotin (Pierce). PDGF was initially dialyzed twice at 4°C for 30 min against PBS in Slide-A-Lyzer MINI Dialysis Devices, 3.5K MWCO (Pierce). Following dialysis, the volume of PDGF solution was determined and its concentration was measured with Nanodrop spectrometer (Thermo Scientific) at 280 nm. Solution of Sulfo-NHS-SS-Biotin in an appropriate volume according to the manufacturer's protocol was added to PDGF and incubated at room temperature for 30 min. Incubation was followed by a 2-min quenching of excess of Sulfo-NHS-SS-Biotin with 2 M glycine. Following biotinylation and quenching, bt-PDGF solution was dialyzed twice at 4°C for 30 min against 0.01 M sodium acetate, pH 4.5. The final concentration was measured and stock solution was stored at −20°C. The extent of bt-PDGF biotinylation was analyzed by ultrafleXtreme™ MALDI-TOF/TOF mass spectrometer (Bruker Daltonics) exactly as previously described 65.

### Generation of fluorescently labeled PDGF

PDGF-BB was labeled with Alexa Fluor 546 carboxylic acid, succinimidyl ester (Invitrogen). PDGF was initially dialyzed against 0.1 M NaHCO_3_ pH 7.3 in Slide-A-Lyzer MINI Dialysis Device, 3.5K MWCO (Pierce). Following dialysis, the solution of Alexa Fluor 546 carboxylic acid, succinimidyl ester was added to PDGF in an appropriate volume according to the manufacturer's protocol and incubated at room temperature for 1 h with shaking. Incubation was followed by 1 h dialysis in darkness against 0.01 M sodium acetate, pH 4.5. Efficiency of coupling was measured according to the manufacturer's protocol and yielded 1.4 Alexa-546 molecules per molecule of PDGF.

### Cell culture and stimulation with PDGF

CCD-1070Sk human normal foreskin fibroblasts were purchased from ATCC (number CRL-2091) and grown in minimum essential eagle (MEM) supplemented with 10% fetal bovine serum (FBS), 2 mM l-glutamine, 100 U/mL penicillin, 100 µg/mL streptomycin (all from Sigma-Aldrich) and maintained in 5% CO_2_ at 37°C. Cells were seeded on 12-mm glass coverslips in a 24-well dish for microscopy or directly in wells of a 24-well dish for western blot experiments. Twenty-four hours before every stimulation, cells were serum-starved in MEM supplemented with 0.2% BSA (Bioshop Canada), 100 U/mL penicillin and 100 µg/mL streptomycin. On the day of stimulation, the medium was exchanged for CO_2_-independent medium (Invitrogen) supplemented with 0.2% BSA, 100 U/mL penicillin and 100 µg/mL streptomycin and the cells were transferred into a 37°C incubator with CO_2_. From this point, all reagents were diluted in CO_2_-independent medium. Thirty minutes before stimulation, cells were pretreated with dynamin or *src* kinase inhibitors or the corresponding controls. MRC-5 human fetal lung fibroblasts (ATCC number CCL-171) were grown and stimulated as described for CCD-1070Sk.

For quantitative microscopy of PDGF trafficking, CCD-1070Sk cells were stimulated with 100 ng/mL of bt-PDGF. This was a minimal amount well detectable by microscopy and permitting quantitative analysis. Following stimulation, cells were transferred on ice and washed twice with ice-cold PBS. Subsequently, cells were incubated on ice for 5 min with glutathione stripping solution (50 mM reduced glutathione, 150 mM NaCl, 70 mM NaOH, 1.25 mM MgSO_4_, 1.25 mM CaCl_2,_ 1 mM EDTA, pH 8.5; modified from 66) to remove biotin from bt-PDGF bound to the extracellular space. Under these conditions, only extracellular disulphide bridges are reduced. Following stripping, the cells were incubated for 5 min on ice with 30 mM iodoacetamide to stop further unwanted reduction of disulphide bonds.

For studies on PDGF signaling using western blot or for microscopic analysis of phospho-STAT3 or cyclin D1, cells were stimulated with 50 ng/mL of unlabeled PDGF for the indicated time periods at 37°C in the presence of inhibitors or the corresponding controls. If needed, transferrin-Alexa647 was added to cells at 20 µg/mL together with bt-PDGF or unlabeled PDGF. For studies using the dynamin mutant, CCD-1070Sk cells were transfected with pEGFP-N1-dynamin2-K44A plasmid using jetPEI (Polyplus Transfection) according to the manufacturer's protocol and after 24 h stimulated with PDGF as described above.

### Immunofluorescence and image quantification

After stimulation with bt-PDGF and stripping of extracellular biotins, cells were rinsed twice for 5 min with ice-cold PBS and fixed with 3% paraformaldehyde in PBS for 12 min at room temperature, followed by a simultaneous permeabilization with 0.1% (w/v) saponin and blocking with 0.2% (w/v) fish gelatin in PBS for 10 min. They were further incubated with appropriate primary and secondary antibodies in 0.01% (w/v) saponin and 0.2% fish gelatin in PBS for 30 min each. Images for qualitative analysis were acquired in 12-bit depth with Leica TCS SP2 microscope equipped with AOBS using 63×/1.4 NA oil immersion objective, 200 Hz speed and 1024 × 1024 pixel resolution. For quantitative analysis, at least ten 12-bit images of each experimental condition were acquired with Zeiss LSM710 microscope using 40×/1.30 oil immersion objective and 1024 × 1024 pixel resolution. Images were imported into MotionTracking/Kalaimoscope (http://www.kalaimoscope.com) 29, 30 to analyze the integral fluorescence of a particular marker in all vesicles (expressed in arbitrary units, AU) and percentage of colocalization between two markers. Error bars on the graphs are standard error of the mean (SEM). The data are representative of three independent experiments.

Immunostaining for nuclear accumulation of phospho-STAT3 or cyclin D1 was performed as follows: after costimulation with 50 ng/mL unlabeled PDGF-BB and 20 µg/mL transferrin-Alexa647, cells were rinsed twice in an ice-cold PBS and fixed with 3% paraformaldehyde in PBS for 30 min at room temperature. Then, the cells were permeabilized in methanol at −20°C for 10 min, blocked with 10% FBS for 20 min and incubated with primary antibodies for 4 h and secondary antibodies together with DAPI for 30 min, both diluted in 1 mg/mL BSA in PBS. In all microscopy figures, single confocal sections are shown. To analyze nuclear accumulation of cyclin D1, images were imported into MBF ImageJ (http://www.macbiophotonics.ca/imagej/). Cell nuclei were identified as regions of interest based on DAPI staining and then a mean gray value of cyclin D1 fluorescence intensity in these regions was measured. Figures were assembled in Photoshop (Adobe) with only linear adjustments.

### Western blot analysis and immunoprecipitation

Following stimulation, cells were transferred on ice and washed twice with ice-cold PBS and lysed in RIPA lysis buffer (150 mM NaCl, 1% NP-40, 0.1% SDS, 0.5% sodium deoxycholate, 50 mM Tris–HCl pH 8.0) in the presence of protease inhibitors (6 µg/mL chymostatin, 0.5 µg/mL leupeptin, 10 µg/mL antipain, 2 µg/mL aprotinin, 0.7 µg/mL pepstatin A and 10 µg/mL 4-amidinophenylmethanesulfonyl fluoride hydrochloride; Sigma-Aldrich, Bioshop Canada) and phosphatase inhibitor cocktail (Sigma-Aldrich), and centrifuged for 10 min at 20 000× ***g***. Protein concentration was measured with microBCA kit (Pierce). Lysates (30 µg of total protein) were boiled in Laemmli sample buffer for 10 min and resolved on 10% polyacrylamide gels followed by a transfer onto nitrocellulose membrane (Whatman). Next, the transfer membrane was incubated with appropriate primary antibodies and subsequently with secondary antibodies for infrared detection. Membranes were scanned on ImageQuant LAS 4000 or LI-COR Odyssey platform. Quantitative analysis of bands detected on LI-COR Odyssey platform was performed with Image Studio Software (LI-COR Biotechnology). The data are representative of three independent experiments.

*Src* activity was analyzed by immunoprecipitation from the lysates of cells non-stimulated or stimulated with 50 ng/mL PDGF in the presence of dynasore, SKI-1, SU6656 or DMSO (cells were pretreated with the inhibitors for 30 min before adding PDGF and the stimulation was performed in the presence of inhibitors). Immunoprecipitation was carried out by overnight incubation of cell lysates with an anti-*src* antibody or non-immune rabbit immunoglobulins at 4°C with constant rotation. Immune complexes were recovered by 2 h incubation with Protein A-agarose beads (Roche) at 4°C with rotation, followed by centrifugation and six washes in IP buffer (50 mM HEPES, 150 mM NaCl, 1 mM EDTA, 1 mM EGTA, 0.1% Triton-X-100, 10% glycerol, pH 7.4). Next, samples were incubated at 95°C for 10 min with Laemmli buffer and subjected to western blot analysis.

### BrdU incorporation

Cell proliferation was tested by BrdU incorporation as a marker of S-phase. Cells were seeded in a 96-well dish with a transparent bottom (Greiner) and serum-starved overnight. Following starvation and pretreatment with inhibitors for 30 min as described above, cells were stimulated with 50 ng/mL PDGF, or 10% serum, or left non-stimulated, in the presence of appropriate inhibitors or corresponding controls for 1 h. After stimulation, cells were washed twice with PBS and placed in a serum-free medium supplemented with 10 μM BrdU for further 24 h. Then, the cells were fixed with 3% paraformaldehyde in PBS for 12 min and DNA was denatured by addition of 2 M HCl for 30 min. Acidic pH was neutralized with 0.1 M borate buffer (pH 8.5) and samples were processed for immunofluorescence as described before 67. Primary mouse anti-BrdU and secondary donkey anti-mouse Alexa-488 antibodies were used. The nuclei were stained with DAPI. Samples were analyzed with Olympus Scan^R/Cell^R system. From each well, 16 images in two channels (BrdU and DAPI) were taken in a random manner. Analysis of obtained images was performed with Olympus Scan^R software. The graphs represent the mean of three independent experiments.

### Quantitative real-time PCR

Cells were seeded in a six-well dish and serum-starved overnight. Following starvation and pretreatment with inhibitors for 30 min as described above, cells were stimulated with 50 ng/mL PDGF in the presence of appropriate inhibitors or corresponding controls for 1 h. After stimulation, cells were washed twice with PBS and placed in a serum-free medium for the indicated time-points. Next, cells were scraped and total RNA was isolated using High Pure RNA Isolation kit (Roche) according to the manufacturer's protocol. The quality of RNA was evaluated by 1% agarose gel electrophoresis. cDNA was synthesized using murine Moloney leukaemia virus reverse transcriptase (Sigma-Aldrich). The following primers were designed using QuantPrime software and synthesized by Sigma-Aldrich: *B2M* (β-2 microglobulin): 5′-TGGAGGCTATCCAGCGTACTC-3′ and 5′-TGAAACCCAGACACATAGCAATTC-3′; *ACTB* (β-actin): 5′-CAGGTCATCACCATTGGCAAT-3′ and 5′-TCTTTGCGGATGTCCACGT-3′; *MYC* (c-Myc): 5′-CGGTGCAGCCGTATTTCTACT-3′ and 5′-GCTCGAATTTCTTCCAGATATCCT-3′; *CCND1* (Cyclin D1): 5′-CTGGAGGTCTGCGAGGAACA-3′ and 5′-TGCAGGCGGCTCTTTTTC-3′ Reactions were performed in triplicates using Kapa SYBR FAST qPCR kit (Kapa Biosystems) according to the manufacturer's protocol. qPCR was performed using a 7900HT Fast Real-Time PCR System (Applied Biosystems). Data quantification was performed using RQ Manager v1.21 and Data Assist v2.0 software (Applied Biosystems). The graphs represent the mean of three independent experiments.

## Supporting Information

**Figure S1: Additional characterization of the experimental system.** A) Adhesion of fluorescently labeled PDGF to glass. Image of CCD-1070Sk cells stimulated with 1 μg/ml PDGF-Alexa 546 for 20 min. Red areas represent extracellular background, while areas with little staining represent cells. Scale bar 20 μm. B) Unspecific inhibition of PDGFR activation by Iminodyn-22. Activation of PDGFR upon stimulation of cells with 50 ng/ml PDGF in the presence of Iminodyn-22 or DMSO, visualized by immunoblotting with phospho-tyrosine (pTYR) antibodies (total STAT3 as loading control). C) Block of endocytosis of PDGF or transferrin elicited by Iminodyn-22. Cells were stimulated with 100 ng/ml PDGF and 20 μg/ml transferrin-Alexa647 for 30 min in a presence of Iminodyn-22 or DMSO. PDGFRβ was visualized by immunostaining. Scale bar 20 μm. D) Lack of STAT3 activation upon low dose of PDGF. Lysates of cells stimulated with 5 ng/ml PDGF were immunoblotted for phospho-tyrosine, phosphoSTAT3, -AKT and total STAT3. E) Dynamics of PDGF-induced STAT3 activation in MRC-5 fibroblasts. Lysates of cells stimulated with PDGF in the presence of DMSO or dynasore were immunoblotted for phosphoSTAT3, -AKT, -ERK1/2 and total STAT3.

**Figure S2: Levels of cyclin D1 upon treatment of cells with dynasore.** A) Immunofluorescence analysis of cyclin D1 nuclear accumulation. CCD-1070Sk cells stimulated with PDGF (50 ng/mL) and transferrin-Alexa647 in the presence of dynasore or DMSO for the indicated times were fixed and immunostained for cyclin D1. NS, serum-starved cells not stimulated with PDGF. Scale bar 20 μm. B) For each experimental condition presented in A, 10 images were analyzed to determine mean intensity values of cyclin D1 fluorescence in the cell nuclei. Error bars are SD.
