# Supplementary material for: Dynamin Inhibitors Impair Endocytosis and Mitogenic Signaling of PDGF
Source: Traffic. 2013 Mar 12;14(6):725–36. doi: 10.1111/tra.12061 (PMC3712465; doi:10.1111/tra.12061)
Supplement: Supplementary file 1 [file tra0014-0725-SD1.doc]

**
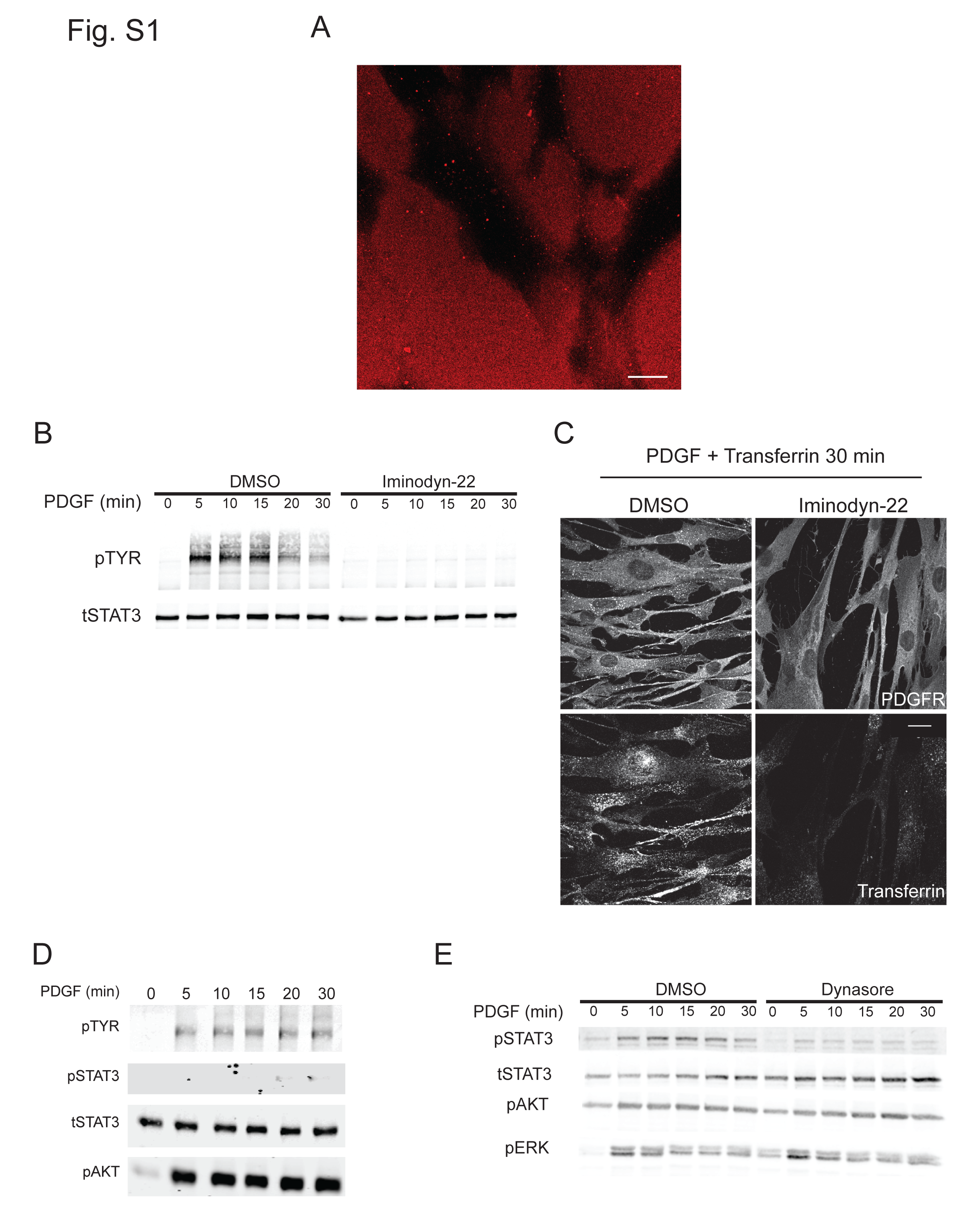
**

**Supplementary Figure 1**

A) Adhesion of fluorescently labeled PDGF to glass.

Image of CCD-1070Sk cells stimulated with 1 µg/ml PDGF-Alexa 546 for 20 min. Red areas represent extracellular background, while areas with little staining represent cells. Scale bar 20 µm.

B) Unspecific inhibition of PDGFR activation by Iminodyn-22.

Activation of PDGFR upon stimulation of cells with 50 ng/ml PDGF in the presence of Iminodyn-22 or DMSO, visualized by immunoblotting with phospho-tyrosine (pTYR) antibodies (total STAT3 as loading control).

C) Block of endocytosis of PDGF or transferrin elicited by Iminodyn-22.

Cells were stimulated with 100 ng/ml PDGF and 20 µg/ml transferrin-Alexa647 for 30 min in a presence of Iminodyn-22 or DMSO. PDGFRβ was visualized by immunostaining. Scale bar 20 µm.

D) Lack of STAT3 activation upon low dose of PDGF.

Lysates of cells stimulated with 5 ng/ml PDGF were immunoblotted for phospho-tyrosine, phosphoSTAT3, -AKT and total STAT3.

E) Dynamics of PDGF-induced STAT3 activation in MRC-5 fibroblasts. Lysates of cells stimulated with PDGF in the presence of DMSO or dynasore were immunoblotted for phosphoSTAT3, -AKT, -ERK1/2 and total STAT3.
