# Supplementary material for: Dynamin Inhibitors Impair Endocytosis and Mitogenic Signaling of PDGF
Source: Traffic. 2013 Mar 12;14(6):725–36. doi: 10.1111/tra.12061 (PMC3712465; doi:10.1111/tra.12061)
Supplement: Supplementary file 2 [file tra0014-0725-SD2.doc]

**
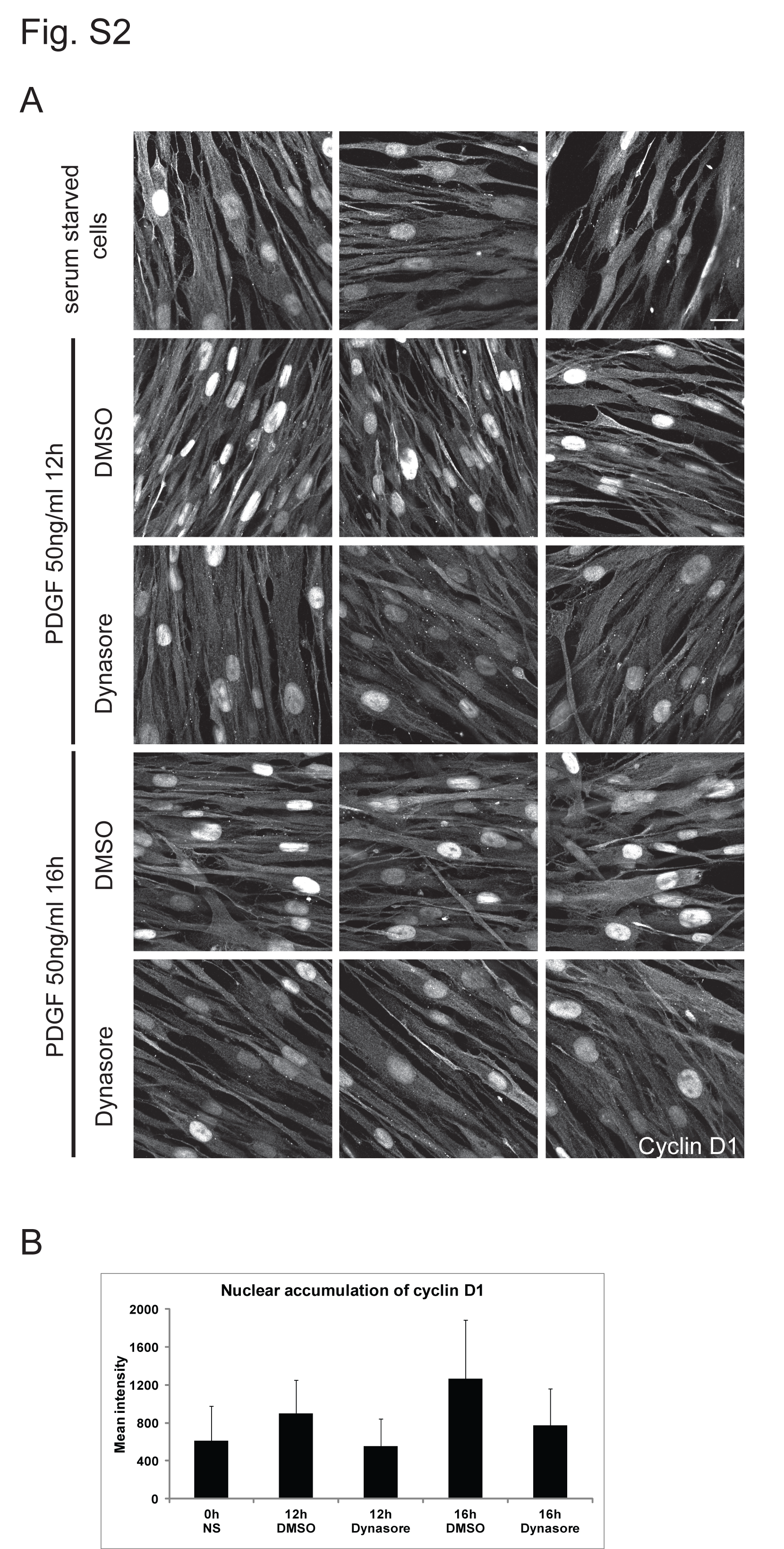
**

**Supplementary Figure 2**

A) Immunofluorescence analysis of cyclin D1 nuclear accumulation. CCD-1070Sk cells stimulated with PDGF (50 ng/ml) and transferrin-Alexa647 in the presence of dynasore or DMSO for the indicated times were fixed and immunostained for cyclin D1. NS, serum-starved cells not stimulated with PDGF. Scale bar 20 µm.

B) For each experimental condition presented in A, 10 images were analyzed to determine mean intensity values of cyclin D1 fluorescence in the cell nuclei. Error bars are SD.
